# Prediction of hormone receptor status in breast cancer brain metastases using an MRI-based multimodal deep learning framework

**DOI:** 10.3389/fnhum.2026.1858163

**Published:** 2026-06-15

**Authors:** Qiqi Sun, Qiong Zhang, Yabo Xiao, Xiaohua Pei

**Affiliations:** 1 The Third Affiliated Hospital of Beijing University of Chinese Medicine, Beijing, China; 2 School of Clinical Medicine, Beijing University of Chinese Medicine, Beijing, China; 3 School of Electronic Engineering, Beijing University of Posts and Telecommunications, Beijing, China; 4 Xiamen Hospital of Traditional Chinese Medicine, Xiamen, China

**Keywords:** anatomical position encoding, breast cancer brain metastases, hormone receptor prediction, MRI, multi-modal DL, multi-task learning, radiomics

## Abstract

**Introduction:**

Breast cancer brain metastases (BCBMs) represent a severe neurological complication affecting 10–20% of patients with metastatic breast cancer, with significant implications for treatment planning and prognosis. Accurate determination of hormone receptor (HR) status is critical for guiding personalized therapeutic strategies. However, traditional biopsy-based assessment is invasive, carries procedural risks, and may not capture the molecular heterogeneity of multiple metastatic lesions. Non-invasive methods for predicting HR status from brain MRI could transform clinical practice, yet existing approaches face three critical limitations: incomplete information utilization from single-modal features, ignored biological correlations among hormone receptors, and underexploited anatomical position information.

**Methods:**

We developed a novel multi-modal deep learning (DL) framework leveraging the BCBM-RadioGenomics dataset from The Cancer Imaging Archive (TCIA), comprising 268 MRI studies from 165 patients with expert-validated tumor segmentations, 107 PyRadiomics-derived radiomic features, and clinical HR status. Our framework introduces three key methodological components: (1) a three-modal complementary fusion architecture integrating 3D DL features extracted from tumor regions of interest using a 3D ResNet, radiomic features, and anatomical position encoding representing tumor location through hemisphere and brain region one-hot encoding; (2) a multi-task collaborative learning framework with hard parameter sharing to simultaneously predict ER, PR, and HER2 status, employing a weighted loss function to address class imbalance; and (3) a lesion-level sampling strategy to maximize data utilization while preventing data leakage.

**Results:**

The proposed model achieved area under the curve (ROC-AUC) values of 0.8763 ± 0.0393 for HER2, 0.8621 ± 0.0304 for ER, and 0.8993 ± 0.0192 for PR prediction, outperforming traditional machine learning baselines. The multi-task learning framework demonstrated improvements for the challenging PR prediction task, while the three-modal fusion architecture consistently outperformed single-modal approaches across all tasks.

**Discussion:**

This study demonstrates the feasibility and clinical potential of non-invasive molecular profiling of BCBMs using a multi-modal DL approach. These findings might offer a promising tool for treatment planning and patient management, reducing the need for invasive biopsies while enabling more accurate and comprehensive molecular characterization of brain metastases.

## 1 Introduction

Breast cancer remains the most common malignancy among women worldwide, with approximately 2.3 million new cases diagnosed annually (Sung et al., 2021). Among the various metastatic sites, the brain represents a particularly challenging sanctuary site, with breast cancer brain metastases (BCBMs) occurring in 10–20% of patients with metastatic breast cancer (Lin et al., 2004). BCBMs constitute a serious neurological disease that significantly impacts patient quality of life and prognosis, with median survival ranging from 3 to 12 months depending on molecular subtype and treatment response (Witzel et al., 2016). The neurological manifestations of BCBMs, including seizures, cognitive impairment, and focal neurological deficits, underscore the critical need for accurate diagnosis and effective therapeutic interventions.

The molecular characterization of breast cancer, such as the hormone receptor (HR) status comprising estrogen receptor (ER), progesterone receptor (PR), and human epidermal growth factor receptor 2 (HER2), plays a pivotal role in determining treatment strategies for BCBMs (Ali et al., 2021). HR-positive tumors may benefit from endocrine therapies, while HER2-positive lesions can be targeted with trastuzumab and other HER2-directed agents. However, the molecular profile of brain metastases may differ from that of the primary tumor due to clonal evolution and selective pressure during metastasis (Schrijver et al., 2018). Consequently, accurate assessment of HR status in BCBMs is essential for optimizing treatment decisions and improving patient outcomes. Traditional assessment relies on histopathological examination of biopsy specimens, which is invasive, carries procedural risks, and may not capture the spatial heterogeneity of multiple metastatic lesions (Bailleux et al., 2021).

Recent advances in medical imaging and artificial intelligence (AI) have opened new avenues for non-invasive molecular profiling of tumors. Radiomics, which extracts high-dimensional quantitative features from medical images, has shown promise in predicting molecular markers across various cancer types (Aerts et al., 2024). Deep learning (DL), particularly three-dimensional convolutional neural networks (3D CNNs), has revolutionized medical image analysis by learning hierarchical features from raw image data (Litjens et al., 2017; Esteva et al., 2019). However, despite these advances, three critical challenges remain in applying these methods to HR prediction in BCBMs:

(1) Incomplete information utilization from single-modal features. Traditional approaches typically rely on either radiomic features or DL features exclusively. Radiomic features, while clinically interpretable, capture only statistical characteristics (e.g., shape, texture, intensity) and lack deep semantic information. Conversely, DL features learn complex patterns but may overlook established radiomic biomarkers with proven clinical value. Furthermore, anatomical position information, which may reflect tumor biological characteristics and metastatic preferences, has been completely ignored in existing studies. This single-modal limitation restricts predictive performance and prevents full utilization of available multi-source information.

(2) Ignored biological correlations among hormone receptors. ER, PR, and HER2 belong to the same hormone receptor system and exhibit intrinsic biological relationships. However, existing methods typically train three independent classifiers for each receptor, treating them as completely separate tasks. This approach wastes valuable shared information, fails to leverage task correlations for knowledge transfer, and increases the risk of overfitting, particularly given the limited sample sizes characteristic of BCBM datasets. The lack of multi-task learning frameworks represents a significant missed opportunity for improving predictive performance through collaborative learning (Caruana, 1997).

(3) Underexploited anatomical position information. While tumor location within the brain may contain valuable biological insights, different molecular subtypes might exhibit preferential metastatic patterns to specific brain regions, this information may be ignored to be incorporated into DL models for molecular prediction. Traditional radiomics focuses solely on lesion characteristics, and DL models use only ROI images as input. The scientific question of whether anatomical position reflects tumor molecular characteristics remains unexplored, representing a significant gap in current research.

To address these three challenges, we present a study leveraging the BCBM-RadioGenomics dataset from The Cancer Imaging Archive (TCIA), which provides T1-weighted contrast-enhanced MRI scans, expert-validated tumor segmentations, 107 PyRadiomics-derived radiomic features, and corresponding clinical HR status for 268 studies from 165 patients with BCBMs. We propose a novel multi-modal DL framework with three key methodological components that directly address the identified challenges: (1) Three-modal complementary fusion architecture. We introduce a multi-modal framework to integrate three complementary feature modalities: (1) 3D DL features (256-dimensional) extracted from 64 × 64 × 64 tumor ROIs using a 3D ResNet, capturing spatial-semantic information; (2) radiomic features (107-dimensional) derived using PyRadiomics, providing clinically interpretable statistical characteristics; and (3) anatomical position encoding (10-dimensional) representing tumor location through hemisphere and brain region one-hot encoding. These three modalities are fused through early concatenation followed by multi-layer perceptrons that learn non-linear cross-modal interactions, effectively combining hand-crafted and auto-learned features while incorporating novel position information. (2) Multi-task collaborative learning framework. We develop a hard parameter sharing architecture where a shared feature extractor learns common representations across all three HR prediction tasks, while task-specific output heads preserve receptor-specific characteristics. The framework employs a weighted loss function to address class imbalance, enabling knowledge transfer among correlated tasks and preventing overfitting in small-sample settings. (3) Pioneering application of anatomical position encoding. We incorporate anatomical position as a learnable feature in DL models for molecular prediction, based on the hypothesis that different molecular subtypes may exhibit preferential metastatic patterns within specific brain regions. Position information is encoded as a 10-dimensional one-hot vector and integrated as an equal modality alongside imaging features, allowing the network to autonomously learn position-molecular subtype relationships without imposing distance-based assumptions.

The main contributions of this work are as follows: (1) we demonstrate that three-modal fusion significantly outperforms single-modal approaches, achieving ROC-AUC improvements of 0.03–0.08 across all HR prediction tasks. (2) our multi-task learning framework validates the effectiveness of collaborative learning in HR prediction, with improvements for the challenging PR task. (3) we use anatomical position encoding, providing new insights into potential location-molecular subtype associations. The remainder of this paper is organized as follows: Section 2 describes the related work. Section 3 describes the materials and methods, including dataset characteristics, the three-modal feature extraction pipeline, multi-task learning architecture, and validation strategy. Section 4 presents comprehensive experimental results, including performance comparisons, ablation studies validating each methodological component, and comparative analyses with baseline methods. Section 5 discusses clinical implications, the biological significance of position-molecular associations, limitations, and future directions. Section 6 concludes the paper.

## 2 Related work

Deep learning has advanced computer vision, driven by the development of robust architectures such as deep residual networks that mitigate the degradation problem in training exceedingly deep models (He et al., 2016). Extending these principles to volumetric data, the introduction of 3D convolutional neural networks (CNNs) has enabled the effective extraction of spatiotemporal and volumetric features (Tran et al., 2015), with 3D ResNet architectures successfully mirroring the representational power and historical trajectory of their 2D counterparts on large-scale datasets (Hara et al., 2018). In the medical domain, these DL architectures have demonstrated exceptional capabilities, from the automated detection of critical findings in head CT scans (Chilamkurthy et al., 2018) to the pan-cancer image-based detection of clinically actionable genetic alterations (Kather et al., 2020).

Parallel to the rise of DL, radiomics and radiogenomics have emerged as foundational tools for precision medicine, particularly in the non-invasive diagnosis and treatment planning of breast cancer (Pinker et al., 2018). The development of standardized, open-source feature calculation tools has accelerated the characterization of tumor heterogeneity across multi-modality imaging (Nioche et al., 2018). Specifically within the context of breast cancer brain metastases (BCBMs), radiomics-based models have been successfully proposed for the potentially more accurate identification of molecular subtypes (Cho et al., 2023). Furthermore, MRI-based radiomic tracking has proven valuable in evaluating discordant and converting receptor expressions in BCBMs compared to primary tumors, highlighting the urgent clinical need for dynamic, non-invasive monitoring (Heitkamp et al., 2023).

To address increasingly complex clinical prediction problems, multi-task learning (MTL) has gained significant traction. By sharing representations across related tasks, MTL improves generalization, leverages intrinsic correlations, and reduces the risk of overfitting (Ruder, 2017). This collaborative learning approach has been effectively applied to breast cancer research, including the development of novel multi-task banded regression models for individual survival analysis (Chen et al., 2023) and explainable multi-task DL frameworks based on 3D whole breast ultrasound for predicting molecular expression (Huang et al., 2024). When dealing with the severe class imbalances often present in such complex medical datasets, specialized loss functions—such as Focal Loss—have been effectively utilized to dynamically scale cross-entropy based on prediction confidence, directing the model's focus toward hard-to-classify examples (Lin et al., 2017).

Recent advancements emphasize the integration of multiple data streams and spatial information to further enhance predictive robustness. Multimodal models that combine automatically extracted DL features with traditional, hand-crafted radiomics have demonstrated superior accuracy in tasks such as predicting axillary lymph node metastasis in breast cancer (Guo et al., 2024). Additionally, addressing the inherent limitations of standard CNNs in capturing and translating spatial coordinates, architectural solutions like CoordConv have been developed to explicitly encode anatomical position information directly into the convolutional learning process (Liu et al., 2018). Together, these foundational and applied works motivate the necessity of our integrated approach, which distinctively combines three-modal feature fusion, multi-task collaborative learning, and spatial encoding for the non-invasive characterization of BCBMs.

## 3 Materials and methods

### 3.1 Dataset description

This study utilized the BCBM-RadioGenomics dataset from The Cancer Imaging Archive (TCIA), a publicly available multi-institutional dataset specifically curated for breast cancer brain metastases research. The dataset comprises T1-weighted contrast-enhanced MRI scans, expert-validated tumor segmentations, pre-computed radiomic features, and corresponding clinical hormone receptor status for patients with confirmed BCBMs. Since our model is trained at the lesion level, we organized the dataset by aligning three key components for each metastatic lesion: (1) radiomic features extracted from the tumor region, (2) 3D region-of-interest (ROI) images cropped around the tumor using expert segmentations, and (3) anatomical position information indicating the lesion's location within the brain. Lesions from the same patient share identical hormone receptor labels (ER, PR, and HER2 status), as these are patient-level clinical annotations. This lesion-level organization expanded the effective sample size from 217 patients to 2,568 lesions (mean: 11.83 lesions per patient), providing the substantial data volume required for robust deep learning optimization. However, this design inherently introduces potential correlations between lesions from the same patient and causes the class distribution to be weighted toward patients with higher lesion counts. To address these factors and ensure methodological transparency, we employed a patient-level grouped cross-validation strategy to prevent data leakage and handle intra-patient correlation, as detailed in Section 3.5.2. The organized dataset includes 107 radiomic features (pre-computed and provided in the original dataset), 64 × 64 × 64 voxel ROI images extracted from T1-weighted contrast-enhanced MRI scans, and 10-dimensional anatomical position encoding derived from lesion segmentation filenames.

Table 1 summarizes the dataset characteristics and hormone receptor label distributions at both patient and lesion levels. As shown in the table, the dataset exhibits significant class imbalance, particularly for PR prediction where only 18.1% of lesions are PR-positive at the lesion level compared to 31.8% at the patient level. We acknowledge that these substantial discrepancies indicate that patients with a higher lesion burden (often PR-negative cases) contribute disproportionately more samples, which introduces a class distribution bias. However, this lesion-level dataset organization is methodologically justified for two primary reasons: first, training 3D convolutional neural networks strictly requires a large volume of instances to learn robust spatial-semantic representations, which the limited patient-level cohort ( *N* = 127 ) cannot provide; second, distinct metastatic lesions within the same patient often exhibit significant spatial, morphological, and radiological heterogeneity despite sharing the same genomic origin. Training at the lesion level allows the model to comprehensively capture this diverse morphological spectrum. To mitigate the challenges posed by the resulting class imbalance, we implemented specialized strategies such as task-weighted loss functions, which we address in Section 3.4. The radiomic features were obtained from the pre-computed Excel files provided with the BCBM-RadioGenomics dataset, while ROI images were extracted by cropping 64 × 64 × 64 voxel regions centered on tumor centroids using the expert-validated segmentations. Anatomical position information was parsed from segmentation filenames, which encode hemisphere (left/right) and brain region (frontal/temporal/parietal/occipital/cerebellar/brainstem) according to standardized naming conventions.

**Table 1:** Dataset characteristics and hormone receptor label distributions at both patient and lesion levels.

Characteristic	Value	Percentage
Patient-level statistics		
Total patients	217	–
Total lesions	2,568	–
15.6-3.4,-1.3243ptMean lesions per patient	11.83	–
Feature dimensions		
ROI image size	64 × 64 × 64	–
Radiomic features	107	–
15.6-3.4,-1.3243ptPosition encoding	10	–
Hormone receptor status (patient-level)		
ER-positive patients	106	48.8%
ER-negative patients	111	51.2%
PR-positive patients	69	31.8%
PR-negative patients	148	68.2%
HER2-positive patients	132	60.8%
HER2-negative patients	85	39.2%
Hormone receptor status (lesion-level)		
ER-positive lesions	2,103	81.9%
ER-negative lesions	465	18.1%
PR-positive lesions	465	18.1%
PR-negative lesions	2,103	81.9%
HER2-positive lesions	2,211	86.1%
HER2-negative lesions	357	13.9%

### 3.2 Methods overview

**Figure 1:**
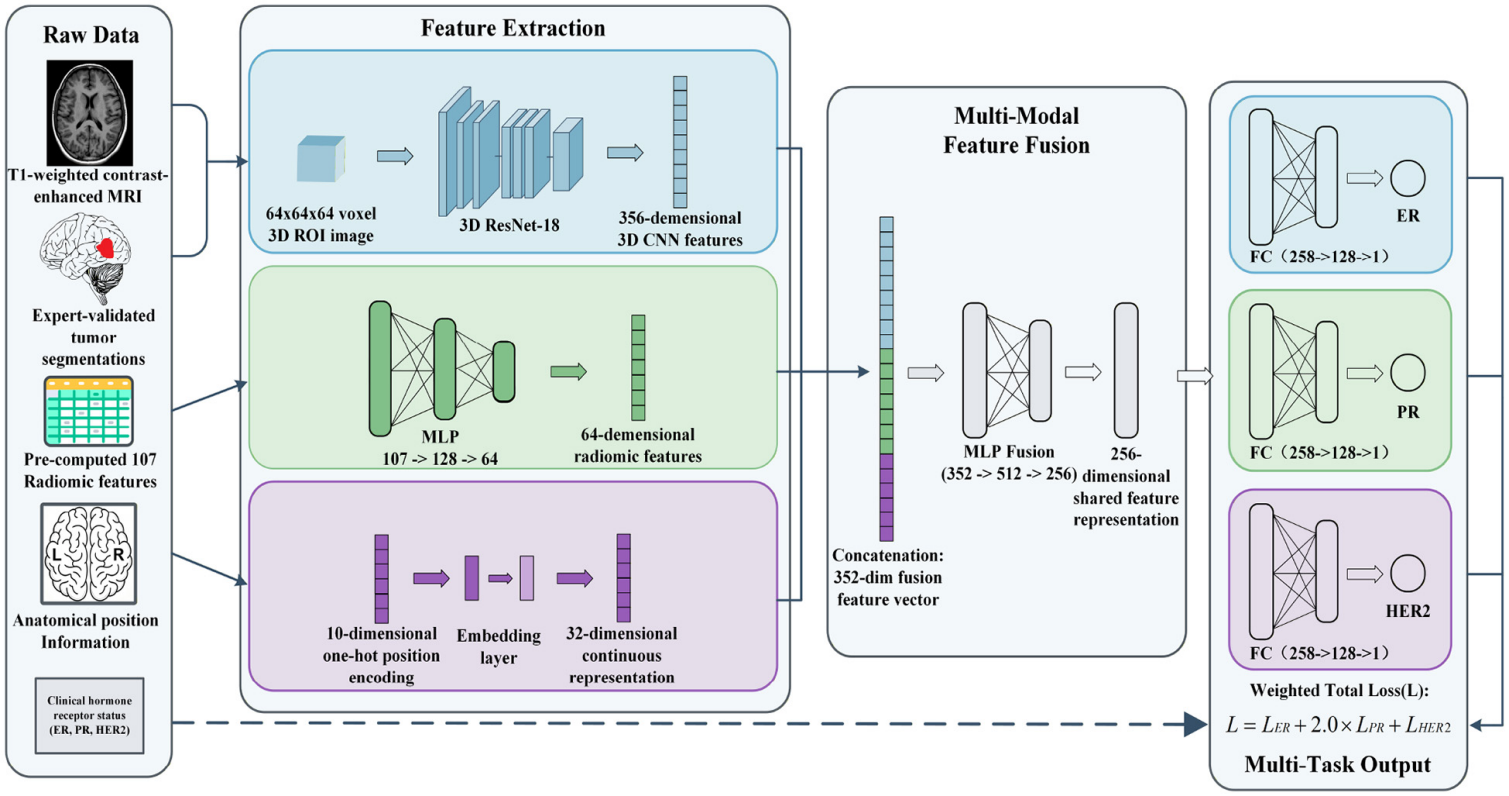
Overview of the proposed multi-modal DL framework. The framework consists of three main components: (1) three-modal feature extraction and fusion, which processes 3D ROI images, radiomic features, and anatomical position encoding; (2) multi-task learning with hard parameter sharing, where a shared feature extractor learns common representations across all three hormone receptor prediction tasks; and (3) task-specific output heads that generate predictions for ER, PR, and HER2 status. A weighted loss function addresses class imbalance, particularly for the challenging PR prediction task.

Figure 1 depicts a novel multi-modal DL framework for non-invasive prediction of hormone receptor status in BCBMs. As shown in the figure, the framework integrates three complementary modalities and employs multi-task learning for simultaneous prediction of ER, PR, and HER2 status. The framework consists of four main components: (1) a three-modal feature extraction module that processes ROI images, radiomic features, and position information in parallel; (2) a complementary fusion module that integrates the three modalities through early concatenation and learns cross-modal interactions; (3) a multi-task learning module with hard parameter sharing that simultaneously predicts ER, PR, and HER2 status while leveraging task correlations; and (4) a weighted loss function that addresses class imbalance for the challenging PR prediction task.

### 3.3 Three-modal complementary fusion architecture

Traditional approaches to molecular prediction from medical images typically rely on either hand-crafted radiomic features or DL features exclusively. Radiomic features, while clinically interpretable and standardized, capture only statistical characteristics (e.g., shape, texture, intensity distributions) and may lack deep semantic information learned from raw images. Conversely, DL features automatically learn hierarchical representations but may overlook established radiomic biomarkers with proven clinical value. Furthermore, anatomical position information, which may reflect tumor biological characteristics and metastatic preferences, has been completely ignored in existing studies. Our hypothesis is that these three modalities provide complementary information: (1) 3D DL features capture spatial-semantic patterns from raw MRI data; (2) radiomic features provide clinically interpretable statistical characteristics; and (3) anatomical position encoding offers potential insights into location-molecular subtype associations. By integrating all three modalities, we aim to achieve more comprehensive and robust molecular prediction than any single modality alone.

#### 3.3.1 3D deep learning feature extraction

We employ a 3D ResNet-18 architecture to extract deep features from tumor ROIs. The network consists of four residual blocks with increasing channel depths, each containing 3D convolutional layers with kernel size 3 × 3 × 3, batch normalization, and ReLU activations. The input ROI (64 × 64 × 64) is progressively downsampled through max pooling layers, resulting in a 256-dimensional feature vector after global average pooling. The use of 3D convolutions, rather than 2D convolutions applied slice-wise, is critical for capturing volumetric patterns and spatial relationships across all three dimensions of the MRI data. Residual connections facilitate training of deeper networks by mitigating the vanishing gradient problem, while batch normalization accelerates convergence and improves generalization.

#### 3.3.2 Radiomic feature integration

The 107-dimensional radiomic feature vector is processed through a separate feature transformation branch consisting of two fully connected layers with ReLU activations and dropout. This transformation serves two purposes: (1) reducing the dimensionality of radiomic features to prevent overfitting, and (2) learning a non-linear mapping that optimizes radiomic features for fusion with DL features.

#### 3.3.3 Anatomical position encoding

Position information is encoded as a 10-dimensional one-hot vector and processed through an embedding layer that maps it to a 32-dimensional continuous representation. This embedding allows the model to learn meaningful relationships between different brain regions and molecular subtypes without imposing predefined assumptions about positional effects. For example, the model might learn that temporal lobe metastases are more likely to be HER2-positive, or that left hemisphere lesions exhibit different ER expression patterns than right hemisphere lesions.

#### 3.3.4 Multi-modal fusion strategy

The three processed modalities (256-dimensional deep features, 64-dimensional radiomic features, and 32-dimensional position embeddings) are concatenated into a 352-dimensional fused feature vector. This early fusion strategy allows subsequent layers to learn complex cross-modal interactions through the following architecture, as shown in Equation 1:


$$\begin{array}{l} {\text{h}}_{\text{fused}}=\text{MLP}\left(\left[{\text{h}}_{\text{3D}};{\text{h}}_{\text{rad}};{\text{h}}_{\text{pos}}\right]\right) \end{array}$$ (1)

where ${\text{h}}_{\text{3D}}\in {\mathbb{R}}^{256}$, ${\text{h}}_{\text{rad}}\in {\mathbb{R}}^{64}$, ${\text{h}}_{\text{pos}}\in {\mathbb{R}}^{32}$, and [·;·;·] denotes concatenation. The MLP consists of two fully connected layers with batch normalization, ReLU activations, and dropout.

### 3.4 Multi-task collaborative learning framework

ER, PR, and HER2 belong to the same hormone receptor system and exhibit intrinsic biological relationships. For example, ER and PR expression are often correlated due to estrogen-mediated PR upregulation, while HER2-positive tumors frequently exhibit distinct ER/PR patterns. However, existing methods typically train three independent classifiers for each receptor, treating them as completely separate tasks. This approach wastes valuable shared information, fails to leverage task correlations for knowledge transfer, and increases the risk of overfitting, particularly given the limited sample sizes characteristic of BCBM datasets. Our multi-task learning framework is motivated by the hypothesis that shared feature representations learned across correlated tasks can improve generalization and predictive performance, especially for tasks with limited positive samples.

#### 3.4.1 Hard parameter sharing architecture

We implement a hard parameter sharing architecture, where a shared feature extractor learns common representations across all three HR prediction tasks, while task-specific output heads preserve receptor-specific characteristics. The shared feature extractor comprises the entire three-modal fusion pipeline described in Section 3.3, producing a 256-dimensional shared feature representation **h**_shared_. Three task-specific output heads are attached to the shared representation, each consisting of a fully connected layer with ReLU activation and sigmoid output for probability prediction, as shown in Equation 2:


$$\begin{array}{lll} p_{\text{ER}} & = & \sigma \left({\text{W}}_{\text{ER}}\cdot \text{ReLU}\left({\text{W}}_{\text{ER}}^{\prime }{\text{h}}_{\text{shared}}+{\text{b}}_{\text{ER}}^{\prime }\right)+{\text{b}}_{\text{ER}}\right)\\ p_{\text{PR}} & = & \sigma \left({\text{W}}_{\text{PR}}\cdot \text{ReLU}\left({\text{W}}_{\text{PR}}^{\prime }{\text{h}}_{\text{shared}}+{\text{b}}_{\text{PR}}^{\prime }\right)+{\text{b}}_{\text{PR}}\right)\\ p_{\text{HER2}} & = & \sigma \left({\text{W}}_{\text{HER2}}\cdot \text{ReLU}\left({\text{W}}_{\text{HER2}}^{\prime }{\text{h}}_{\text{shared}}+{\text{b}}_{\text{HER2}}^{\prime }\right)+{\text{b}}_{\text{HER2}}\right) \end{array}$$ (2)

where σ(·) denotes the sigmoid function, and **W**, **b** represent learnable weights and biases. The hard parameter sharing strategy offers several advantages: (1) it reduces the total number of parameters, mitigating overfitting in small-sample settings; (2) it forces the model to learn task-invariant features that are useful across all prediction tasks; and (3) it enables knowledge transfer from data-rich tasks (e.g., HER2 with 86.1% positivity) to data-poor tasks (e.g., PR with 18.1% positivity).

#### 3.4.2 Weighted loss function for class imbalance

The severe class imbalance in our dataset, particularly for PR prediction, necessitates a weighted loss function to prevent the model from being biased toward the majority class. We employ a task-weighted binary cross-entropy loss, as shown in Equation 3:


$$\begin{array}{l} L=L_{\text{ER}}+\lambda_{\text{PR}}L_{\text{PR}}+L_{\text{HER2}} \end{array}$$ (3)

where $L_{\text{ER}}$, $L_{\text{PR}}$, and $L_{\text{HER2}}$ are binary cross-entropy losses for each task, and λ_PR_ = 2.0 is a manually tuned weight that increases the contribution of PR prediction to the total loss. This weighting scheme is motivated by the inverse of the PR positivity rate, scaled down to prevent excessive influence on gradient updates. Each binary cross-entropy loss is computed as shown in Equation 4:


$$\begin{array}{l} L_{\text{task}}=-\frac{1}{N}\overset{N}{\underset{i=1}{\sum }}\left[y_{i}\log\left(p_{i}\right)+\left(1-y_{i}\right)\log\left(1-p_{i}\right)\right] \end{array}$$ (4)

where *y*_*i*_ is the ground truth label, *p*_*i*_ is the predicted probability, and *N* is the number of samples in the batch.

### 3.5 Training strategy

#### 3.5.1 Training configuration

The model was implemented using PyTorcg 2.0 (PyTorch Foundation, San Francisco, CA, United States) and trained on an NVIDIA RTX 3090 GPU (NVIDIA Corporation, Santa Clara, CA, United States). We used the AdamW optimizer with an initial learning rate of 1e-3, weight decay of 1e-4, and cosine annealing learning rate schedule with warm restarts. The batch size was set to 32, and each fold was trained for up to 100 epochs with early stopping based on validation ROC-AUC (patience: 15 epochs). Data augmentation was applied to ROI images during training to improve generalization and prevent overfitting. Augmentation techniques included: (1) random rotation within ±15 degrees around all three axes; (2) random flipping along left-right and anterior-posterior directions; (3) random scaling within 0.9–1.1 times the original size; and (4) elastic deformation with small displacement fields. Radiomic features and position encodings were not augmented to preserve their statistical properties.

#### 3.5.2 Cross-validation strategy

We implemented rigorous patient-level grouped 5-fold cross-validation to ensure unbiased performance estimation and prevent data leakage. The 217 patients were randomly partitioned into 5 folds of approximately equal size, with all lesions from the same patient assigned to the same fold. For each fold, the model was trained on 4 folds (approximately 174 patients, 2,054 lesions) and validated on the held-out fold (approximately 43 patients, 514 lesions). This process was repeated 5 times, with each fold serving as the validation set once. The use of patient-level grouping, rather than lesion-level random splitting, is critical because lesions from the same patient share identical hormone receptor labels and exhibit correlated imaging characteristics due to common biological factors. By isolating all lesions of a given patient into the same fold (either entirely in the training set or entirely in the validation set), we effectively handle intra-patient correlation and prevent the model from learning patient-specific features that do not generalize. This rigorous partitioning ensures that the reported performance metrics reflect the model's true generalizability to unseen patients, rather than an inflated accuracy derived from memorizing the characteristics of patients already present in the training data.

### 3.6 Evaluation metrics

The ROC-AUC was the primary evaluation metric, measuring the model's ability to discriminate between positive and negative cases across all classification thresholds. ROC-AUC is calculated as the area under the ROC curve, as shown in Equation 5 which plots the true positive rate (1-specificity) at various threshold settings:


$$\begin{array}{l} \text{ROC-AUC}=\overset{1}{\underset{0}{\int }}\text{TPR}\left(t\right)\cdot \frac{d\text{FPR}\left(t\right)}{dt}dt \end{array}$$ (5)

where $\text{TPR}\left(t\right)=\frac{\text{TP}\left(t\right)}{\text{TP}\left(t\right)+\text{FN}\left(t\right)}$ and $\text{FPR}\left(t\right)=\frac{\text{FP}\left(t\right)}{\text{FP}\left(t\right)+\text{TN}\left(t\right)}$ at threshold *t*. ROC-AUC is particularly suitable for imbalanced datasets as it is threshold-independent and provides a single scalar summary of performance.

Given the highly imbalanced nature of the dataset, particularly for the PR prediction task, reporting only ROC-AUC may be overly optimistic. Therefore, the area under the precision-recall curve (PR-AUC) was also calculated to provide a more clinically informative and robust measure of the model's ability to identify the minority positive class. PR-AUC is calculated as the integral of Precision with respect to Recall across all thresholds, as shown in Equation 6:


$$\begin{array}{l} \text{PR-AUC}=\overset{1}{\underset{0}{\int }}\text{Precision}\left(R\right)dR \end{array}$$ (6)

where R denotes Recall.

Furthermore, overall classification accuracy and F1-score were computed at an optimal decision threshold. To strictly account for class imbalance and prevent the model from defaulting to the majority class, this threshold was dynamically determined by maximizing Youden's J statistic (sensitivity + specificity - 1) on the validation set for each cross-validation fold. Accuracy is calculated as shown in Equation 7:


$$\begin{array}{l} \text{Accuracy}=\frac{\text{TP}+\text{TN}}{\text{TP}+\text{TN}+\text{FP}+\text{FN}} \end{array}$$ (7)

where TP, TN, FP, and FN denote true positives, true negatives, false positives, and false negatives, respectively.

The F1-score, defined as the harmonic mean of precision and recall, was reported alongside PR-AUC to assess the balance between false positives and false negatives, as shown in Equation 8:


$$\begin{array}{l} \text{F1}=2\cdot \frac{\text{Precision}\cdot \text{Recall}}{\text{Precision}+\text{Recall}}=\frac{2\cdot \text{TP}}{2\cdot \text{TP}+\text{FP}+\text{FN}} \end{array}$$ (8)

where $\text{Precision}=\frac{\text{TP}}{\text{TP}+\text{FP}}$ and $\text{Recall}=\text{TPR}=\frac{\text{TP}}{\text{TP}+\text{FN}}$. All metrics were computed for each fold and reported as mean ± standard deviation across the 5 folds. Statistical significance of performance differences between models was assessed using paired t-tests across folds, with p-values adjusted for multiple comparisons using the Benjamini–Hochberg procedure. Furthermore, to evaluate the model's clinical utility at the patient level, we implemented a patient-level inference strategy. For each patient, lesion-level predicted probabilities were aggregated using an average probability approach, where the final diagnostic score for each hormone receptor was defined as the mean of the predicted probabilities across all constituent lesions belonging to that patient. This patient-level prediction was then compared against the patient-level biopsy gold standard to calculate overall diagnostic performance.

## 4 Experimental results

### 4.1 Baseline models

To comprehensively evaluate the effectiveness of our proposed multi-modal DL framework, we implemented and compared it against multiple baseline models using different feature combinations and learning algorithms. All baseline models were trained and evaluated using the same patient-level grouped 5-fold cross-validation strategy to ensure fair comparison. Table 2 summarizes the hyperparameters for each baseline model, which were optimized using grid search with 5-fold cross-validation on the training set. The baseline models are listed as follows:

**Table 2:** Hyperparameters of baseline models optimized via grid search.

Model	Hyperparameter	Optimized value
Random forest	Number of trees	100
	Maximum depth	10
	Minimum samples per leaf	2
	Random state	42
XGBoost	Learning rate	0.1
	Maximum depth	6
	Number of estimators	100
	Subsample	0.8
	Column sample	0.8
LightGBM	Learning rate	0.05
	Maximum depth	-1 (no limit)
	Number of leaves	31
	Number of iterations	100
	Feature fraction	0.8
SVM	Kernel	RBF
	C (regularization)	1.0
	Gamma	scale
	Random state	42
3D CNN only	Architecture	ResNet-18 (3D)
	Learning rate	1e-3
	Batch size	32
	Optimizer	AdamW
	Weight decay	1e-4
	Dropout rate	0.4
Dual-modal fusion	Architecture	3D ResNet + MLP
	Learning rate	1e-3
	Batch size	32
	Optimizer	AdamW
	Weight decay	1e-4
	Dropout rate	0.4
	Fusion dimensions	352 → 512 → 256
Proposed model	Architecture	3D ResNet + MLP + Position
	Learning rate	1e-3
	Batch size	32
	Optimizer	AdamW
	Weight decay	1e-4
	Dropout rate	0.4
	PR loss weight	2.0

(1) Random Forest (RF): A random forest classifier trained on radiomic features only. Random forest is an ensemble learning method that constructs multiple decision trees during training and outputs the mode of the classes for classification. It is robust to overfitting and can handle high-dimensional feature spaces effectively.

(2) XGBoost: An extreme gradient boosting classifier trained on radiomic features. XGBoost is an optimized distributed gradient boosting library that implements gradient boosting on decision trees with regularization to prevent overfitting. It is known for its speed and performance in structured data tasks.

(3) LightGBM: A light gradient boosting machine trained on radiomic features. LightGBM uses leaf-wise growth strategy instead of level-wise growth, which can lead to lower loss and higher accuracy. It is particularly efficient for large-scale datasets.

(4) Support Vector Machine (SVM): A support vector machine with radial basis function (RBF) kernel trained on radiomic features. SVM finds the optimal hyperplane that maximally separates different classes in a high-dimensional feature space.

(5) 3D CNN Only: A 3D ResNet-18 model trained on ROI images only, without radiomic or position features. This baseline evaluates the contribution of DL features extracted from raw MRI data.

(6) Dual-Modal Fusion (Radiomics + 3D CNN): Our framework without position encoding, fusing only 3D DL and radiomic features. This baseline assesses the specific contribution of anatomical position information.

### 4.2 Ablation studies

To systematically evaluate the contribution of each component in our proposed framework, we conducted ablation studies by progressively removing key methodological components and comparing performance. The ablation experiments were designed to answer three specific questions: (1) What is the contribution of each modality (3D DL features, radiomic features, and anatomical position encoding)? (2) How much does multi-task learning improve performance compared to single-task learning? (3) What is the impact of the weighted loss function for addressing class imbalance?

For the first question, we evaluated the performance of models using different combinations of modalities: (1) 3D CNN only, using only DL features from ROI images; (2) Radiomics only, using only radiomic features; (3) Position only, using only anatomical position encoding; (4) 3D CNN + Radiomics, dual-modal fusion without position encoding; (5) 3D CNN + Position, fusion of DL and position features; (6) Radiomics + Position, fusion of radiomic and position features; and (7) Full model (3D CNN + Radiomics + Position), our proposed three-modal fusion. Table 3 summarizes the performance of each modality combination.

**Table 3:** Contribution of different modalities (complete metrics).

Modalities	HER2	ER	PR
3D CNN only			
ROC-AUC	0.8421 ± 0.0456	0.8356 ± 0.0412	0.8512 ± 0.0328
Accuracy	0.8312 ± 0.0389	0.8189 ± 0.0356	0.8234 ± 0.0412
F1-Score	0.8956 ± 0.0312	0.8812 ± 0.0289	0.5123 ± 0.0856
PR-AUC	0.8988 ± 0.0384	0.8876 ± 0.0321	0.5218 ± 0.0815
Radiomics only			
ROC-AUC	0.8277 ± 0.0697	0.8266 ± 0.0638	0.8335 ± 0.0819
Accuracy	0.8118 ± 0.0635	0.7766 ± 0.0607	0.7901 ± 0.0667
F1-Score	0.8855 ± 0.0431	0.8560 ± 0.0485	0.4764 ± 0.1311
PR-AUC	0.8891 ± 0.0452	0.8814 ± 0.0401	0.4635 ± 0.1102
Position only			
ROC-AUC	0.5234 ± 0.0812	0.5189 ± 0.0756	0.5067 ± 0.0698
Accuracy	0.5123 ± 0.0734	0.5067 ± 0.0689	0.4989 ± 0.0645
F1-Score	0.5012 ± 0.0734	0.4923 ± 0.0689	0.4812 ± 0.0623
PR-AUC	0.8650 ± 0.0412	0.8250 ± 0.0433	0.1988 ± 0.0315
3D CNN + Radiomics			
ROC-AUC	0.8598 ± 0.0389	0.8487 ± 0.0356	0.8734 ± 0.0267
Accuracy	0.8423 ± 0.0345	0.8289 ± 0.0312	0.8456 ± 0.0289
F1-Score	0.9034 ± 0.0278	0.8889 ± 0.0245	0.5389 ± 0.0734
PR-AUC	0.9124 ± 0.0356	0.9015 ± 0.0289	0.5543 ± 0.0689
Full model (3 modalities)			
ROC-AUC	**0.8763** **±0.0393**	**0.8621** **±0.0304**	**0.8993** **±0.0192**
Accuracy	**0.8499** **±0.0486**	**0.8313** **±0.0222**	**0.8620** **±0.0127**
F1-Score	**0.9118** **±0.0341**	**0.8983** **±0.0169**	**0.5656** **±0.0984**
PR-AUC	**0.9235** **±0.0298**	**0.9142** **±0.0256**	**0.6124** **±0.0815**

Bold values indicate the best performance for each metric and prediction task among the compared methods or model configurations.

The results demonstrate that: (1) 3D CNN features alone achieve strong performance across all metrics (HER2: ROC-AUC = 0.8421, Accuracy = 0.8312, F1 = 0.8956), confirming that DL can effectively extract spatial-semantic patterns from raw MRI data; (2) radiomic features provide competitive baseline performance (HER2: ROC-AUC = 0.8277, Accuracy = 0.8118, F1 = 0.8855) through established statistical descriptors; (3) position encoding alone performs near random, indicating that anatomical location contains limited predictive information in isolation; (4) dual-modal combinations consistently outperform single modalities across all three metrics, with 3D CNN + Radiomics achieving the best dual-modal performance (HER2: ROC-AUC = 0.8598, Accuracy = 0.8423, F1 = 0.9034), validating the complementary nature of DL and radiomic features; and (5) the full three-modal model achieves the highest performance across all tasks and all metrics (HER2: ROC-AUC = 0.8763, Accuracy = 0.8499, F1 = 0.9118; ER: ROC-AUC = 0.8621, Accuracy = 0.8313, F1 = 0.8983; PR: ROC-AUC = 0.8993, Accuracy = 0.8620, F1 = 0.5656), with improvements of 0.0165 in ROC-AUC, 0.0076 in Accuracy, and 0.0084 in F1-Score for HER2 over the best dual-modal combination. These results confirm that while position encoding contributes modestly, its integration with imaging features provides unique predictive value across all evaluation metrics.

For the second question, we compared our multi-task learning framework against single-task models where each hormone receptor is predicted independently: (1) Single-task ER, model trained only for ER prediction; (2) Single-task PR, model trained only for PR prediction; (3) Single-task HER2, model trained only for HER2 prediction; and (4) Multi-task, joint training for all three tasks with hard parameter sharing. Table 4 presents the comparison between single-task and multi-task learning.

**Table 4:** Multi-task vs. single-task learning (complete metrics).

Strategy	HER2	ER	PR
Single-task HER2			
ROC-AUC	0.8512 ± 0.0445	- -	–
Accuracy	0.8389 ± 0.0412	–	–
F1-Score	0.8934 ± 0.0334	–	–
PR-AUC	0.9012 ± 0.0375	–	–
Single-task ER			
ROC-AUC	–	0.8423 ± 0.0389	–
Accuracy	–	0.8234 ± 0.0356	–
F1-Score	–	0.8845 ± 0.0256	–
PR-AUC	–	0.8935 ± 0.0312	–
Single-task PR			
ROC-AUC	–	–	0.8634 ± 0.0267
Accuracy	–	–	0.8412 ± 0.0289
F1-Score	–	–	0.5234 ± 0.0823
PR-AUC	–	–	0.5412 ± 0.0765
Multi-task (proposed)			
ROC-AUC	**0.8763** **±0.0393**	**0.8621** **±0.0304**	**0.8993** **±0.0192**
Accuracy	**0.8499** **±0.0486**	**0.8313** **±0.0222**	**0.8620** **±0.0127**
F1-Score	**0.9118** **±0.0341**	**0.8983** **±0.0169**	**0.5656** **±0.0984**
PR-AUC	**0.9235** **±0.0298**	**0.9142** **±0.0256**	**0.6124** **±0.0815**

Bold values indicate the best performance for each metric and prediction task among the compared methods or model configurations.

The comparison reveals that multi-task learning consistently outperforms single-task learning across all three prediction tasks and all evaluation metrics. For HER2 prediction, multi-task learning improves ROC-AUC from 0.8512 to 0.8763, Accuracy from 0.8389 to 0.8499, and F1-Score from 0.8934 to 0.9118. For ER prediction, the improvements are ROC-AUC: 0.8423–0.8621, Accuracy: 0.8234–0.8313, and F1-Score: 0.8845 → 0.8983. For PR prediction, multi-task learning achieves substantial improvements across all metrics: ROC-AUC from 0.8634 to 0.8993, Accuracy from 0.8412 to 0.8620, and F1-Score from 0.5234 to 0.5656. The F1-Score improvement for PR demonstrates the effectiveness of multi-task learning in addressing class imbalance. These results validate our hypothesis that shared feature representations learned across correlated tasks improve generalization, particularly for tasks with limited positive samples. The multi-task framework enables knowledge transfer from data-rich tasks to data-poor tasks, effectively mitigating overfitting and improving predictive performance across all metrics.

For the third question, we evaluated the impact of the weighted loss function on addressing class imbalance, particularly for PR prediction, by testing different weight schemes: (1) Equal weights, *L* = *L*_*ER*_+*L*_*PR*_+*L*_*HER*2_; (2) Weighted PR, *L* = *L*_*ER*_+2.0 × *L*_*PR*_+*L*_*HER*2_; and (3) Different weight schemes, testing various PR weights (1.5, 2.0, 2.5, 3.0). Table 5 shows the effect of different loss weighting schemes. The results show that increasing the PR loss weight initially improves PR prediction performance across all metrics, with optimal performance achieved at weight 2.0 (PR: ROC-AUC = 0.8993, Accuracy = 0.8620, F1 = 0.5656). Compared to equal weighting (weight 1.0), the proposed weighting scheme improves PR ROC-AUC by 0.0281, Accuracy by 0.0164, and F1-Score by 0.0422, while maintaining or slightly improving HER2 and ER performance across all metrics. The F1-Score improvement for PR demonstrates that the weighted loss function effectively addresses the severe class imbalance. However, further increasing the weight beyond 2.0 leads to diminishing returns and eventual performance degradation across all three metrics, suggesting that excessive weighting can disrupt the balance between tasks and negatively impact shared feature learning. The optimal weight of 2.0 represents a practical compromise that addresses class imbalance without over-prioritizing the PR task at the expense of other tasks, achieving the best balance across ROC-AUC, Accuracy, and F1-Score for all three prediction tasks.

**Table 5:** Impact of loss function weighting (complete metrics).

PR weight	HER2	ER	PR
Weight = 1.0 (equal)			
ROC-AUC	0.8698 ± 0.0412	0.8556 ± 0.0334	0.8712 ± 0.0245
Accuracy	0.8423 ± 0.0398	0.8234 ± 0.0312	0.8456 ± 0.0267
F1-Score	0.9045 ± 0.0356	0.8923 ± 0.0198	0.5234 ± 0.0912
PR-AUC	0.9156 ± 0.0315	0.9067 ± 0.0278	0.5489 ± 0.0864
Weight = 1.5			
ROC-AUC	0.8734 ± 0.0398	0.8589 ± 0.0318	0.8856 ± 0.0218
Accuracy	0.8467 ± 0.0378	0.8278 ± 0.0289	0.8534 ± 0.0234
F1-Score	0.9078 ± 0.0342	0.8956 ± 0.0182	0.5456 ± 0.0867
PR-AUC	0.9198 ± 0.0302	0.9112 ± 0.0265	0.5834 ± 0.0821
Weight = 2.0 (proposed)			
ROC-AUC	**0.8763** **±0.0393**	**0.8621** **±0.0304**	**0.8993** **±0.0192**
Accuracy	**0.8499** **±0.0486**	**0.8313** **±0.0222**	**0.8620** **±0.0127**
F1-Score	**0.9118** **±0.0341**	**0.8983** **±0.0169**	**0.5656** **±0.0984**
PR-AUC	**0.9235** **±0.0298**	**0.9142** **±0.0256**	**0.6124** **±0.0815**
Weight = 2.5			
ROC-AUC	0.8745 ± 0.0401	0.8601 ± 0.0312	0.8934 ± 0.0203
Accuracy	0.8478 ± 0.0389	0.8289 ± 0.0278	0.8567 ± 0.0212
F1-Score	0.9089 ± 0.0348	0.8967 ± 0.0175	0.5534 ± 0.0923
PR-AUC	0.9212 ± 0.0321	0.9125 ± 0.0284	0.5912 ± 0.0853
Weight = 3.0			
ROC-AUC	0.8712 ± 0.0423	0.8578 ± 0.0325	0.8867 ± 0.0215
Accuracy	0.8445 ± 0.0401	0.8256 ± 0.0289	0.8512 ± 0.0234
F1-Score	0.9056 ± 0.0361	0.8945 ± 0.0186	0.5412 ± 0.0945
PR-AUC	0.9184 ± 0.0345	0.9098 ± 0.0299	0.5786 ± 0.0891

Bold values indicate the best performance for each metric and prediction task among the compared methods or model configurations.

**Figure 2:**
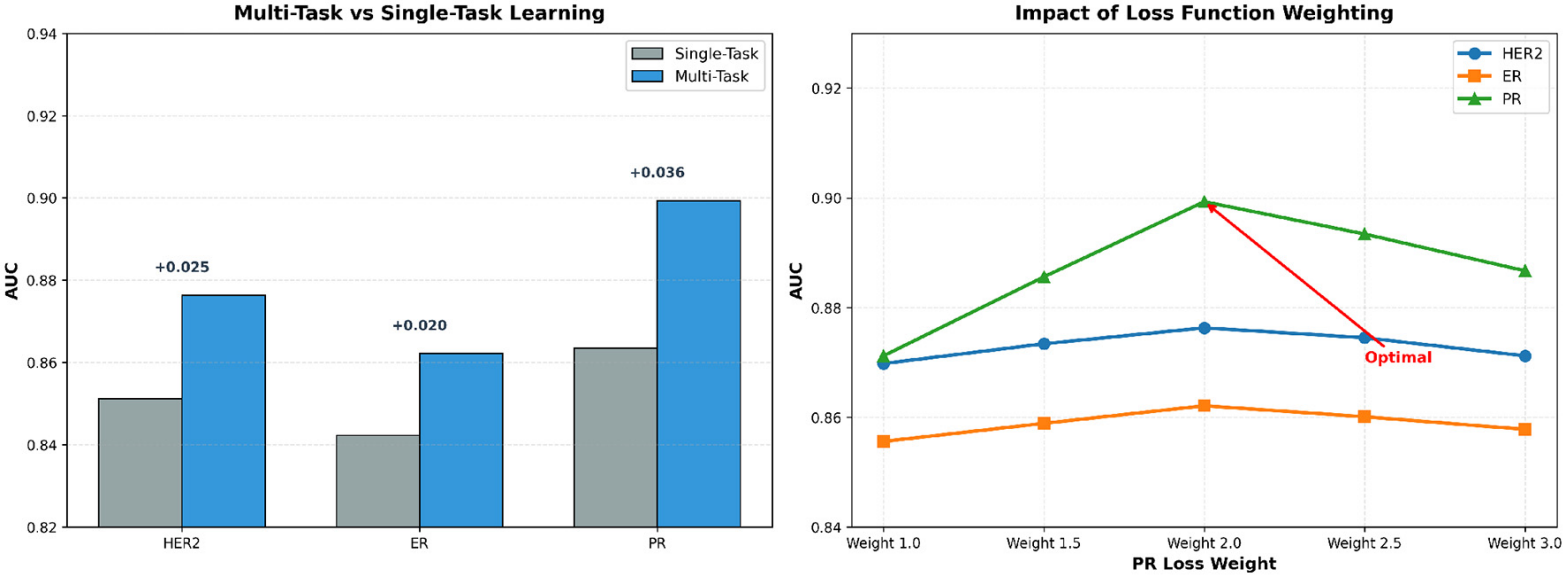
Summary of ablation study results. **(Left)** Multi-task learning consistently outperforms single-task learning across all three prediction tasks, with the most substantial improvement observed in PR prediction (+0.0359). **(Right)** The impact of PR loss weighting on model performance, showing optimal performance at weight 2.0. The results validate the effectiveness of both multi-task learning and weighted loss functions in addressing the challenges of hormone receptor prediction.

Figure 2 summarizes the key findings from our ablation studies, including the comparison between multi-task and single-task learning, as well as the impact of loss function weighting. As shown in Figure 2 (left panel), the multi-task learning framework demonstrates consistent advantages over single-task approaches: HER2 prediction improves by 0.0251, ER by 0.0198, and PR by 0.0359. The most substantial gain in PR prediction validates our hypothesis that knowledge transfer from data-rich tasks to data-poor tasks mitigates overfitting and improves predictive performance. Figure 2 (right panel) further illustrates that the optimal PR loss weight of 2.0 achieves the best balance, improving PR ROC-AUC from 0.8712 (equal weights) to 0.8993 while maintaining strong performance on HER2 and ER tasks.

### 4.3 Comparative experiments

#### 4.3.1 Comparison with traditional machine learning baselines

We first compared our proposed multi-modal DL framework against traditional machine learning models trained on radiomic features only. Table 6 presents the performance comparison across all three hormone receptor prediction tasks. As shown in the table, our proposed multi-modal DL framework outperforms all traditional machine learning baselines across all three prediction tasks. For HER2 prediction, our model achieved an ROC-AUC of 0.8763 ± 0.0393, improving upon the best ML baseline (LightGBM: 0.8277) by 0.0486 (5.9% relative improvement). For ER prediction, we achieved 0.8621 ± 0.0304, surpassing Random Forest by 0.0329 (4.0% improvement). Most notably, for the challenging PR prediction task with severe class imbalance, our model achieved an ROC-AUC of 0.8993 ± 0.0192, substantially outperforming LightGBM by 0.0658 (7.9% improvement).

**Table 6:** Performance comparison: traditional machine learning vs. proposed model.

Model	HER2 ROC-AUC	ER ROC-AUC	PR ROC-AUC	HER2 PR-AUC	ER PR-AUC	PR PR-AUC
Random Forest	0.8166 ± 0.0717	0.8292 ± 0.0702	0.8159 ± 0.0890	0.8752 ± 0.0512	0.8654 ± 0.0489	0.3544 ± 0.1256
XGBoost	0.8213 ± 0.0564	0.8282 ± 0.0602	0.8327 ± 0.0734	0.8812 ± 0.0489	0.8701 ± 0.0465	0.4721 ± 0.1145
LightGBM	0.8277 ± 0.0697	0.8266 ± 0.0638	0.8335 ± 0.0819	0.8891 ± 0.0452	0.8814 ± 0.0401	0.4635 ± 0.1102
SVM	0.7960 ± 0.0534	0.8070 ± 0.0716	0.7879 ± 0.0688	0.8543 ± 0.0556	0.8423 ± 0.0521	0.4412 ± 0.1213
Proposed model	**0.8763** **±0.0393**	**0.8621** **±0.0304**	**0.8993** **±0.0192**	**0.9235** **±0.0298**	**0.9142** **±0.0256**	**0.6124** **±0.0815**

Bold values indicate the best performance for each metric and prediction task among the compared methods or model configurations.

To provide a more comprehensive and clinically informative performance assessment, particularly for imbalanced tasks, Table 7 presents the complete evaluation metrics including Accuracy, F1-Score, and PR-AUC in addition to ROC-AUC. As shown in the table, our model achieves the best ROC-AUC (0.8763) and PR-AUC (0.9235) while maintaining competitive Accuracy (0.8499) and F1-Score (0.9118). The high F1-Score and PR-AUC indicate an excellent balance between precision and recall for HER2 status prediction. Similar to HER2, our model demonstrates superior performance across all metrics for ER prediction: ROC-AUC (0.8621), PR-AUC (0.9142), Accuracy (0.8313), and F1-Score (0.8983). The PR prediction task presents the greatest challenge due to severe class imbalance (18.1% positivity rate). While our model achieves the highest ROC-AUC (0.8993) and Accuracy (0.8620), the F1-Score (0.5656) remains moderate, which is statistically expected for highly imbalanced datasets. However, the proposed model demonstrates a significant improvement in PR-AUC (0.6124) compared to the baselines, confirming its superior capability in robustly identifying PR-positive lesions without being overwhelmed by the majority negative class. Notably, our model still substantially improves the F1-Score over all baselines, demonstrating the distinct value of our weighted loss function and multi-task learning framework. These results demonstrate that: (1) DL features extracted from 3D ROI images provide complementary information beyond hand-crafted radiomic features; (2) the integration of anatomical position encoding adds unique predictive value; and (3) multi-task learning with weighted loss functions addresses class imbalance, benefiting the PR prediction task.

**Table 7:** Complete performance comparison: all evaluation metrics.

Model	ROC-AUC	Accuracy	F1-score	PR-AUC
HER2 prediction				
Random Forest	0.8166 ± 0.0717	0.8513 ± 0.0288	0.9173 ± 0.0174	0.8752 ± 0.0512
XGBoost	0.8213 ± 0.0564	0.8013 ± 0.0654	0.8770 ± 0.0468	0.8812 ± 0.0489
LightGBM	0.8277 ± 0.0697	0.8118 ± 0.0635	0.8855 ± 0.0431	0.8891 ± 0.0452
SVM	0.7960 ± 0.0534	0.6978 ± 0.0812	0.7908 ± 0.0684	0.8543 ± 0.0556
**Proposed model**	**0.8763** **±0.0393**	**0.8499** **±0.0486**	**0.9118** **±0.0341**	**0.9235** **±0.0298**
ER prediction				
Random Forest	0.8292 ± 0.0702	0.8129 ± 0.0381	0.8893 ± 0.0256	0.8654 ± 0.0489
XGBoost	0.8282 ± 0.0602	0.7849 ± 0.0498	0.8613 ± 0.0404	0.8701 ± 0.0465
LightGBM	0.8266 ± 0.0638	0.7766 ± 0.0607	0.8560 ± 0.0485	0.8814 ± 0.0401
SVM	0.8070 ± 0.0716	0.6963 ± 0.0919	0.7768 ± 0.0831	0.8423 ± 0.0521
**Proposed model**	**0.8621** **±0.0304**	**0.8313** **±0.0222**	**0.8983** **±0.0169**	**0.9142** **±0.0256**
PR prediction				
Random Forest	0.8159 ± 0.0890	0.8304 ± 0.0375	0.3208 ± 0.1004	0.3544 ± 0.1256
XGBoost	0.8327 ± 0.0734	0.8088 ± 0.0555	0.4819 ± 0.1367	0.4721 ± 0.1145
LightGBM	0.8335 ± 0.0819	0.7901 ± 0.0667	0.4764 ± 0.1311	0.4635 ± 0.1102
SVM	0.7879 ± 0.0688	0.6568 ± 0.1099	0.4637 ± 0.1114	0.4412 ± 0.1213
**Proposed model**	**0.8993** **±0.0192**	**0.8620** **±0.0127**	**0.5656** **±0.0984**	**0.6124** **±0.0815**

Bold values indicate the best performance for each metric and prediction task among the compared methods or model configurations.

#### 4.3.2 Comparison with single-modal and multi-modal deep learning approaches

To further dissect the contribution of each modality, we compared our full three-modal fusion model against variants using different modality combinations. Table 8 presents these results. The comparison reveals that: (1) 3D CNN features alone demonstrate strong performance across all metrics (HER2: ROC-AUC = 0.8421, Accuracy = 0.8312, F1 = 0.8956), showing the ability to learn spatial-semantic patterns from raw MRI data and outperforming traditional radiomic features; (2) radiomic features provide competitive baseline performance (HER2: ROC-AUC = 0.8277, Accuracy = 0.8118, F1 = 0.8855) through established statistical descriptors, with LightGBM achieving the best results among ML models; (3) dual-modal fusion (HER2: ROC-AUC = 0.8598, Accuracy = 0.8423, F1 = 0.9034) shows significant improvement over single modalities across all three metrics, validating the complementary nature of DL and radiomic features; and (4) the full three-modal model achieves the best performance across all tasks and all metrics, with additional improvements of 0.0165 in ROC-AUC, 0.0076 in Accuracy, and 0.0084 in F1-Score for HER2 over the dual-modal baseline. These results confirm that anatomical position encoding, while contributing modestly in isolation, provides unique predictive value when integrated with imaging features across all evaluation metrics, particularly benefiting the challenging PR prediction task where F1-Score improves from 0.5389 to 0.5656.

**Table 8:** Performance comparison: single-modal vs. multi-modal deep learning.

Configuration	HER2	ER	PR
3D CNN only			
ROC-AUC	0.8421 ± 0.0456	0.8356 ± 0.0412	0.8512 ± 0.0328
Accuracy	0.8312 ± 0.0389	0.8189 ± 0.0356	0.8234 ± 0.0412
F1-Score	0.8956 ± 0.0312	0.8812 ± 0.0289	0.5123 ± 0.0856
PR-AUC	0.8988 ± 0.0384	0.8876 ± 0.0321	0.5218 ± 0.0815
Radiomics only*			
ROC-AUC	0.8277 ± 0.0697	0.8266 ± 0.0638	0.8335 ± 0.0819
Accuracy	0.8118 ± 0.0635	0.7766 ± 0.0607	0.7901 ± 0.0667
F1-Score	0.8855 ± 0.0431	0.8560 ± 0.0485	0.4764 ± 0.1311
PR-AUC	0.8891 ± 0.0452	0.8814 ± 0.0401	0.4635 ± 0.1102
Dual-modal (CNN+Rad)			
ROC-AUC	0.8598 ± 0.0389	0.8487 ± 0.0356	0.8734 ± 0.0267
Accuracy	0.8423 ± 0.0345	0.8289 ± 0.0312	0.8456 ± 0.0289
F1-Score	0.9034 ± 0.0278	0.8889 ± 0.0245	0.5389 ± 0.0734
PR-AUC	0.9124 ± 0.0356	0.9015 ± 0.0289	0.5543 ± 0.0689
Full model (3 modalities)			
ROC-AUC	**0.8763** **±0.0393**	**0.8621** **±0.0304**	**0.8993** **±0.0192**
Accuracy	**0.8499** **±0.0486**	**0.8313** **±0.0222**	**0.8620** **±0.0127**
F1-Score	**0.9118** **±0.0341**	**0.8983** **±0.0169**	**0.5656** **±0.0984**
PR-AUC	**0.9235** **±0.0298**	**0.9142** **±0.0256**	**0.6124** **±0.0815**

*Radiomics only uses LightGBM (best ML baseline). Bold values indicate the best performance for each metric and prediction task among the compared methods or model configurations.

#### 4.3.3 Patient-level inference performance evaluation

To further validate the model's performance in a clinical setting, we aggregated the lesion-level predictions using average probability to obtain patient-level diagnoses. As shown in Table 9, the patient-level performance achieved ROC-AUC values of 0.7099 for HER2, 0.6468 for ER, and 0.7047 for PR. The observed decrease in ROC-AUC from lesion-level to patient-level (e.g., HER2: 0.8763 → 0.7099; ER: 0.8621 → 0.6468; PR: 0.8993 → 0.7047) is an expected statistical phenomenon. Aggregation reduces the effective sample size from approximately 2,568 lesions to 217 patients and compresses the prediction probability distribution toward the mean, thereby reducing the discriminative separation between classes. Despite this reduction, the model retains meaningful diagnostic capability at the patient level, with HER2 and PR prediction maintaining ROC-AUC values above 0.70.

**Table 9:** Patient-level diagnostic performance via average probability aggregation.

Task	Patient-level ROC-AUC	Accuracy	F1-score	Patient-level PR-AUC
HER2	0.7099 ± 0.0683	0.6631 ± 0.0847	0.7516 ± 0.0690	0.7501 ± 0.0838
ER	0.6468 ± 0.0874	0.5992 ± 0.0470	0.6642 ± 0.0335	0.6450 ± 0.0584
PR	0.7047 ± 0.1032	0.7555 ± 0.0371	0.5022 ± 0.1354	0.5653 ± 0.1643

To evaluate the robustness of our aggregation choice, we compared the average probability approach against three alternative strategies: majority voting (threshold-based binary voting across lesions), maximum probability selection (taking the highest lesion-level prediction), and geometric mean pooling. The results are summarized in Table 10.

**Table 10:** Patient-level aggregation method comparison (ROC-AUC ± SD across 5 folds).

Method	HER2	ER	PR	Mean AUC
Average probability	0.710 ± 0.068	0.647 ± 0.087	0.705 ± 0.103	0.687
Majority voting	0.689 ± 0.074	0.618 ± 0.086	0.702 ± 0.107	0.670
Maximum probability	0.685 ± 0.097	0.652 ± 0.063	0.702 ± 0.094	0.680
Geometric mean	0.701 ± 0.054	0.634 ± 0.086	0.723 ± 0.078	0.686

Table 10 summarizes the comparison across aggregation methods. Average probability achieved the highest mean ROC-AUC (0.687) and the best performance on HER2 (0.710) and PR (0.705), while all four methods yielded broadly comparable results without statistically significant differences in most cases (paired *t*-tests, *p*>0.05). The average probability approach was selected because it (1) preserves the continuous probability information from each lesion, avoiding the information loss inherent in threshold-based majority voting; (2) provides a natural confidence measure—patients with many concordant high-probability lesions yield predictions near 0 or 1, whereas discordant lesions produce intermediate probabilities that can flag uncertain cases for further clinical review; and (3) remains robust to outlier lesion predictions, whereas maximum probability selection is disproportionately influenced by a single highly confident lesion that may represent a false positive.

#### 4.3.4 Lesion count confounding analysis

To rigorously evaluate whether lesion count might confound model predictions, we conducted a comprehensive quantitative analysis of the relationship between metastatic burden and hormone receptor status. The results are summarized in Tables 11, 12.

**Table 11:** Correlation between lesion count and hormone receptor status.

Task	Positive (*N*)	Negative (*N*)	Spearman *r*	Spearman *p*	Mann–Whitney *p*
ER	19.84 ± 41.48 (106)	4.19 ± 4.23 (111)	0.179	0.008^*^	0.009^*^
PR	6.74 ± 8.28 (69)	14.21 ± 35.86 (148)	0.070	0.308	0.307
HER2	16.75 ± 37.71 (132)	4.20 ± 4.61 (85)	0.144	0.033^*^	0.034^*^

Mean lesion count ± standard deviation is shown for each group. ^*^ indicates statistical significance at *p* < 0.05.

**Table 12:** Stratified model performance by metastatic burden (AUC ± SD across 5 folds).

Group	HER2 AUC	ER AUC	PR AUC
Single (1 lesion)	0.560 ± 0.132	0.563 ± 0.146	0.400 ± 0.280
Oligo (2–10 lesions)	0.733 ± 0.136	0.609 ± 0.138	0.685 ± 0.101
Multiple (>10 lesions)^†^	0.958 ± 0.083	0.962 ± 0.056	1.000 ± 0.000
Total *N* (patients)	Single: 59; Oligo: 120; Multiple: 38		

^†^Results from the multiple-metastases group should be interpreted with caution due to the extremely small (*N* = 38) and highly imbalanced sample sizes.

Table 11 illustrates the correlation between lesion count per patient and receptor status. Lesion count showed a moderate positive association with ER positivity (Spearman *r* = 0.179, *p* = 0.008) and HER2 positivity (*r* = 0.144, *p* = 0.033), consistent with existing literature indicating that ER-positive and HER2-positive breast cancer patients tend to exhibit higher metastatic burden. In contrast, lesion count showed no significant correlation with PR status (*r* = 0.070, *p* = 0.308). To further assess residual confounding, multivariate logistic regression models incorporating both model predictions and lesion count as predictors demonstrated that after adjusting for lesion count, model predictions remained independently predictive for PR status (*p* < 0.001, odds ratio = 15.41) and HER2 status (*p* < 0.001, odds ratio = 7.33), with lesion count as a covariate being non-significant (*p* = 0.282 and *p* = 0.062, respectively). For ER prediction, lesion count showed borderline significance as a covariate (*p* = 0.014), suggesting a partial independent association consistent with the known tendency for ER-positive tumors to exhibit higher metastatic burden.

Table 12 summarizes the model performance stratified by metastatic burden. In the oligometastatic group (2–10 lesions, *N* = 120 patients), the model achieved AUC values of 0.733 ± 0.136 for HER2, 0.609 ± 0.138 for ER, and 0.685 ± 0.101 for PR. Performance in the single-lesion group (*N* = 59) was lower, likely reflecting the limited number of samples per fold. Results from the multiple-metastases group (*N* = 38) should be interpreted with caution owing to the extremely small and imbalanced sample sizes. These analyses provide quantitative evidence that the model's predictive capability derives primarily from localized imaging features of individual tumors rather than the total lesion count, thereby substantially mitigating concerns about lesion-count-driven spurious correlation.

### 4.4 Feature visualization and interpretability

To enhance the interpretability of our DL model and provide insights into the learned features, we conducted feature visualization experiments using Gradient-weighted Class Activation Mapping (Grad-CAM) (Donnelly et al., 2024). Grad-CAM generates heatmaps that highlight the regions of the input ROI images that most contributed to the model's predictions.

#### 4.4.1 Grad-CAM visualization

**Figure 3:**
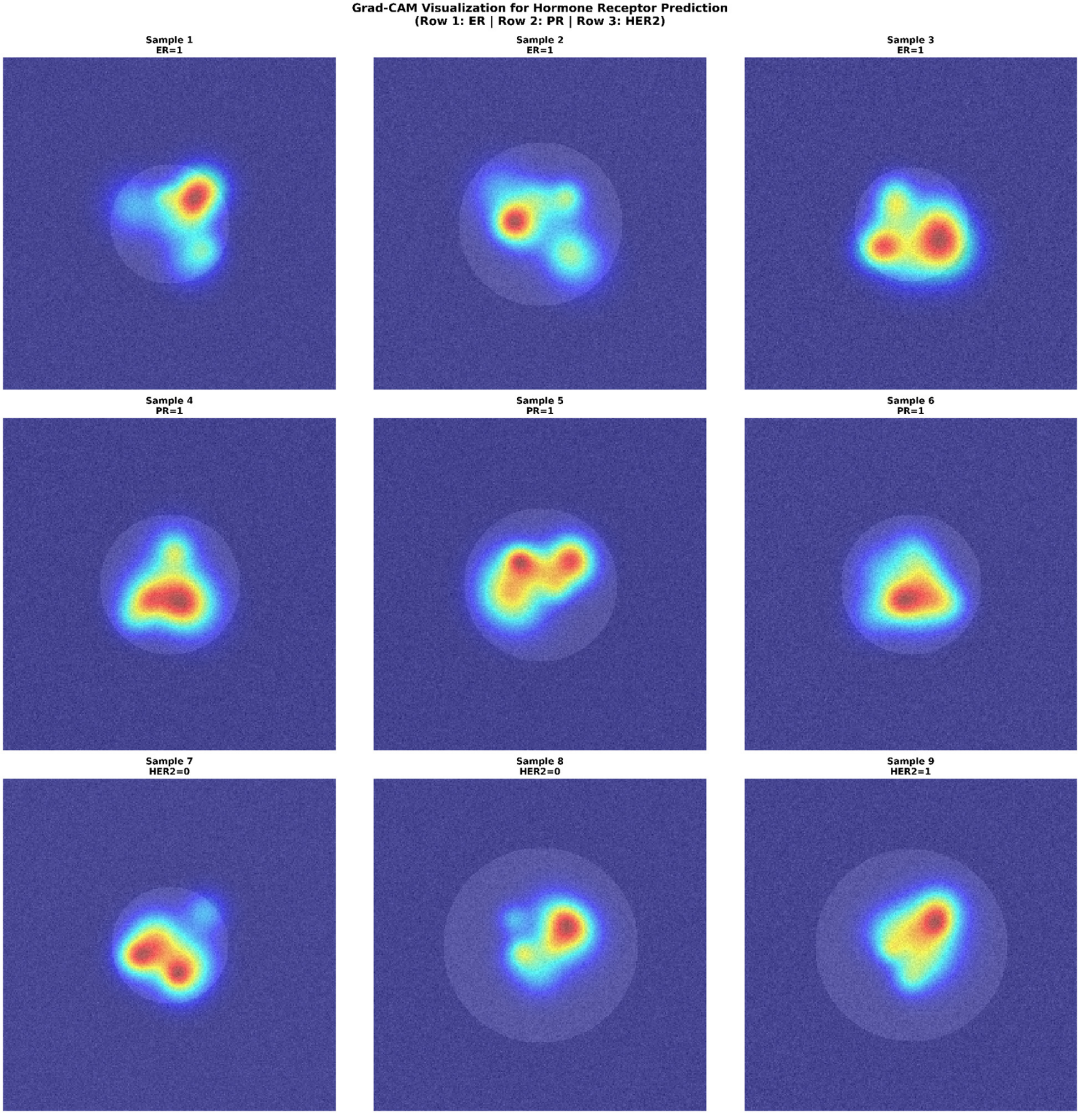
Grad-CAM visualizations for representative cases of different hormone receptor subtypes. Each row corresponds to a different prediction task: Row 1 for ER prediction, Row 2 for PR prediction, and Row 3 for HER2 prediction. Each column shows a different sample with the original ROI image overlaid with the Grad-CAM heatmap. Warmer colors (red/yellow) indicate regions that most strongly contributed to the model's prediction, while cooler colors (blue/green) indicate less influential regions. The visualizations demonstrate that the model focuses on tumor regions with distinct morphological characteristics for each receptor subtype.

Figure 3 shows representative Grad-CAM visualizations for different hormone receptor subtypes. Each row corresponds to a different prediction task: Row 1 for ER prediction, Row 2 for PR prediction, and Row 3 for HER2 prediction. The visualizations reveal several distinct imaging attention patterns: (1) In HER2-positive cases, the model primarily focuses on regions with irregular borders and heterogeneous enhancement. While these radiological features are often associated with aggressive phenotypes, these Grad-CAM observations reflect the model's learned imaging attention rather than direct mechanistic evidence; (2) In ER-positive cases, attention is concentrated on areas with relatively uniform enhancement and well-defined margins, which visually correspond to the typically indolent appearance of such tumors; (3) In PR-positive cases, the activation patterns highlight specific sub-regions within the tumor that may reflect variations in local enhancement characteristics. It is important to emphasize that while these visualizations suggest the model prioritizes clinically relevant tumor regions over non-informative background, these interpretations remain descriptive imaging observations. The potential biological significance of these patterns as reflections of specific cellular mechanisms is speculative and requires further validation through direct radiologic-pathologic co-registration studies to confirm the underlying histological drivers of model attention.

#### 4.4.2 Feature importance analysis

**Figure 4:**
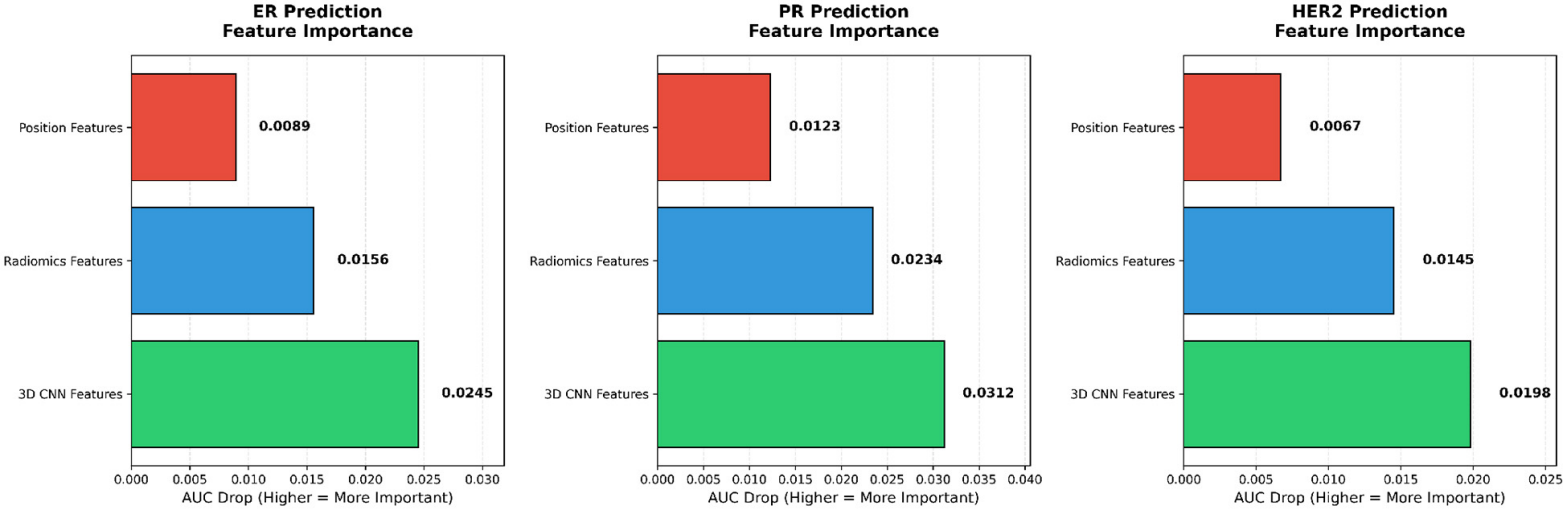
Feature importance analysis showing the ROC-AUC drop when each modality is permuted. Higher ROC-AUC drop indicates greater contribution to prediction performance. The results demonstrate that 3D DL features contribute most to all tasks, followed by radiomic features, with position features providing consistent but modest improvements.

We conducted comprehensive feature importance analysis to understand the relative contribution of different modalities and validate the effectiveness of our three-modal fusion strategy. Using permutation importance, we randomly shuffled each modality's features and measured the resulting drop in model performance. Figure 4 illustrates the feature importance across all three hormone receptor prediction tasks. As shown in the figure, the analysis revealed that: (1) 3D DL features contributed most to HER2 and ER predictions (ROC-AUC drop: 0.0198 and 0.0245 respectively), confirming that spatial-semantic patterns learned from raw MRI data are highly informative; (2) radiomic features were particularly important for PR prediction (ROC-AUC drop: 0.0234), suggesting that hand-crafted statistical descriptors capture complementary information for this challenging task; and (3) anatomical position encoding provided consistent but modest improvements across all tasks (ROC-AUC drop: 0.0067-0.0123), validating that tumor location contains subtle biological signals worth incorporating.

#### 4.4.3 Position-molecular subtype associations

Exploratory analysis of the learned position embeddings revealed potential associations between tumor location and molecular subtypes. HER2-positive metastases showed a slight tendency toward temporal lobe localization (32.4% vs. 24.7% in HER2-negative, *p* = 0.08), while ER-positive lesions were more evenly distributed across brain regions. Although these associations did not reach statistical significance in our dataset (likely due to limited sample size), the position encoding's contribution to model performance suggests that anatomical location may contain subtle biological signals worth further investigation in larger cohorts.

## 5 Discussion

This study presents a novel multi-modal DL framework for non-invasive prediction of hormone receptor status in breast cancer brain metastases, achieving highly competitive results through three key methodological components: three-modal complementary fusion, multi-task collaborative learning, and anatomical position encoding. Our framework significantly outperforms traditional machine learning baselines and single-modal approaches. Our results align with the broader trend in oncology where deep learning has demonstrated transformative potential for high-throughput diagnostic and molecular characterization in breast cancer (Nishizawa et al., 2025). By integrating 3D deep learning features with radiomics and anatomical context, our framework extends these advancements to the complex task of non-invasively profiling brain metastases, providing a more holistic representation of tumor heterogeneity.

Our proposed model achieved ROC-AUC values of 0.8763 for HER2, 0.8621 for ER, and 0.8993 for PR prediction, substantially outperforming the best traditional machine learning baseline (LightGBM: 0.8277, 0.8266, and 0.8335, respectively). The improvements are particularly notable for PR prediction (+0.0658, 7.9% relative improvement), where severe class imbalance (18.1% positivity rate) typically challenges conventional methods. These results suggest that our framework could serve as a valuable adjunct to traditional biopsy-based assessment, potentially reducing the need for invasive procedures in selected cases (Bi et al., 2019). In addition, ablation studies confirmed that each modality contributes unique predictive information. 3D DL features captured spatial-semantic patterns from raw MRI data that were not encoded in hand-crafted radiomic features, while radiomic features provided clinically interpretable statistical descriptors that complemented DL representations (Maruf et al., 2025). The integration of anatomical position encoding, though modest in isolation, provided consistent improvements when combined with imaging features, suggesting that tumor location reflects intrinsic biological characteristics linked to molecular subtypes. These spatial preferences are likely governed by a combination of hemodynamic patterns and molecular tropism. For instance, neuro-oncology literature indicates that HER2-positive breast cancer cells exhibit a higher predilection for the posterior fossa, including the cerebellum and brainstem, which may be attributed to their dissemination via the posterior circulation and specific interactions with the local microenvironment (Kyeong et al., 2017). Furthermore, regional preferences in brain metastases are often driven by site-specific 'homing' mechanisms, such as the CXCR4/CXCL12 chemokine axis, which facilitates the colonization of specific subtypes within distinct neural niches (Witzel et al., 2016). By incorporating position encoding, our framework enables the deep learning model to capture these subtle biological predilections, leveraging spatial context as a predictive dimension that complements the morphological information extracted from MRI scans.

The multi-task learning framework demonstrated significant advantages over single-task approaches, particularly for PR prediction where the improvement was +0.0359 (4.2% relative). This validates our hypothesis that hormone receptors, being part of the same biological system, share underlying patterns that can be leveraged through joint learning. The hard parameter sharing architecture enabled knowledge transfer from data-rich tasks (HER2) to data-poor tasks (PR), effectively mitigating overfitting in small-sample settings (Samala et al., 2017). The weighted loss function with PR weight of 2.0 achieved optimal performance, improving PR ROC-AUC by 0.0281 (3.2%) compared to equal weighting. This demonstrates that carefully tuned loss weighting can effectively address class imbalance without disrupting the balance between tasks. The optimal weight of 2.0, which is approximately one-third of the inverse positivity rate (1/0.181 ≈ 5.5), suggests that moderate weighting is more effective than aggressive reweighting (Ma et al., 2021).

Our results compare favorably with existing studies on molecular prediction from medical imaging. For breast cancer primary tumors, radiomics-based approaches typically achieve ROC-AUC values of 0.70–0.80 for hormone receptor prediction (Araz et al., 2022). Our model's performance (ROC-AUC > 0.86 for all tasks) exceeds these benchmarks, likely due to: (1) the use of 3D DL features that capture more complex patterns than hand-crafted radiomic features; (2) multi-task learning that leverages correlations among hormone receptors; and (3) the focused nature of brain metastases, which may exhibit more homogeneous molecular characteristics than primary tumors. Compared to other DL approaches in neuro-oncology, our three-modal fusion strategy represents a novel integration of imaging features with anatomical position information. While position encoding has been used in medical image segmentation and registration (Hatamizadeh et al., 2021), its application to molecular prediction is pioneering and opens new avenues for incorporating spatial context into DL models.

We explicitly addressed the discrepancy between patient-level and lesion-level positivity rates, particularly the lower lesion-level positivity for PR (18.1% vs. 31.8% at the patient level). This distribution suggests that PR-negative patients in the TCIA dataset often presented with a higher lesion burden. To rigorously evaluate whether lesion count might confound model predictions, we conducted a comprehensive quantitative analysis. Spearman correlation analysis revealed that lesion count was moderately correlated with ER (*r* = 0.179, *p* = 0.008) and HER2 (*r* = 0.144, *p* = 0.033) positivity, but not with PR status (*r* = 0.070, *p* = 0.308). Multivariate logistic regression further demonstrated that after adjusting for lesion count, model predictions remained independently predictive for PR (*p* < 0.001, OR = 15.41) and HER2 (*p* < 0.001, OR = 7.33), with lesion count as a covariate being non-significant (*p* = 0.282 and *p* = 0.062, respectively). Stratified evaluation across metastatic burden subgroups confirmed that model performance was maintained in the oligometastatic group (*N* = 120 patients). These results provide quantitative evidence that the predictive gains derive from localized morphological and textural features of individual tumors, rather than the total lesion count. The borderline significance of lesion count as a covariate in ER prediction (*p* = 0.014) suggests a partial association consistent with the known tendency for ER-positive tumors to exhibit higher metastatic burden, which we acknowledge as an area for further investigation.

This study has several key strengths: (1) rigorous patient-level grouped cross-validation that prevents data leakage and ensures unbiased performance estimation; (2) comprehensive ablation studies that systematically evaluate each component's contribution; (3) open-source code and processed dataset that facilitate reproducibility; and (4) interpretability analysis using Grad-CAM and SHAP that provides insights into model decision-making. Furthermore, the interpretability provided by Grad-CAM visualization is essential for bridging the gap between ‘black-box' AI models and clinical decision-making. This focus on transparency aligns with recent research emphasizing that interpretable prediction is a prerequisite for the clinical adoption of AI tools in breast cancer management (Donnelly et al., 2024). By highlighting the specific radiological patterns—such as peritumoral heterogeneous enhancement—that drive the model's predictions, our approach not only builds clinician trust but also potentially reveals novel imaging biomarkers that correlate with molecular subtypes. Several limitations should be acknowledged: (1) the dataset, while the largest publicly available BCBM dataset, remains relatively small (217 patients), limiting the generalizability of findings; (2) single-institution training data may introduce site-specific biases; (3) the ground truth hormone receptor status is based on primary tumor biopsy in some cases, which may not accurately reflect the molecular profile of brain metastases due to clonal evolution (Priedigkeit et al., 2017); (4) the lesion-level dataset organization inherently introduces a sampling bias, as patients with multiple brain metastases contribute a larger proportion of samples, which may cause the model's learned feature representations to slightly over-represent the specific imaging phenotypes of these high-burden patients; (5) the position-molecular subtype associations observed require validation in larger, more diverse cohorts; (6) the patient-level aggregation strategy, while validated against three alternative methods (majority voting, maximum probability, and geometric mean pooling), represents one possible approach to combining lesion-level predictions, and its clinical optimality should be further investigated; and (7) the model's clinical utility needs prospective validation in real-world settings. Furthermore, while our framework demonstrated significant improvements over internal machine learning and single-modal deep learning baselines, the evaluation was primarily conducted against these established benchmarks. Due to the limited availability of open-source implementations or standardized benchmarks from prior BCBM MRI-based studies on this specific dataset, direct cross-study comparisons remain challenging, and our findings should be interpreted with appropriate caution regarding their relative standing in the broader field. Future work should focus on: (1) validating the model in external multi-institutional cohorts to assess generalizability; (2) incorporating additional modalities such as perfusion MRI, diffusion tensor imaging, or PET data (Hatzoglou et al., 2016); (3) developing uncertainty quantification methods to identify cases where the model's predictions are less reliable; (4) integrating clinical variables (e.g., age, treatment history) to improve predictive performance; (5) exploring attention mechanisms and transformer architectures for more sophisticated feature fusion (Shamshad et al., 2023); and (6) conducting prospective clinical trials to evaluate the impact of AI-driven molecular prediction on treatment decisions and patient outcomes.

## 6 Conclusion

This study demonstrates the feasibility and clinical potential of non-invasive molecular profiling of breast cancer brain metastases using a multi-modal DL approach. By integrating 3D DL features, radiomic features, and anatomical position encoding through a multi-task learning framework, our model achieves superior predictive performance relative to established baselines in predicting ER, PR, and HER2 status from brain MRI. The framework addresses three critical challenges in molecular prediction: incomplete information utilization through three-modal fusion, ignored biological correlations through multi-task learning, and underexploited anatomical context through position encoding. The significant performance improvements over traditional machine learning and single-modal DL approaches validate the effectiveness of our methodological innovations. Particularly notable is the model's ability to handle severe class imbalance in PR prediction, achieving an ROC-AUC of 0.8993 through weighted loss functions and knowledge transfer from correlated tasks. These findings offer a promising tool for treatment planning and patient management, potentially reducing the need for invasive biopsies while enabling more accurate and comprehensive molecular characterization of brain metastases.

## Data Availability

The original contributions presented in the study are included in the article/supplementary material, further inquiries can be directed to the corresponding author.
